# Segmentation and morphometry of intracranial internal carotid artery calcification in relation to brain atrophy

**DOI:** 10.1007/s00234-026-03918-9

**Published:** 2026-03-14

**Authors:** Xiao Xu, Nikhil N. Chaudhari, Phoebe Imms, Nahian F. Chowdhury, Fangyun C. Liu, Jorge A. Solis Galvan, Bavrina Bigjahan, Grant Schleifer, Maria Ashna, Blake Hannagan, Giuseppe Barisano, Daniel K. Cummings, Daniel Eid Rodriguez, Paul L. Hooper, Edmond Seabright, Randall C. Thompson, Benjamin C. Trumble, Michael D. Gurven, Jonathan Stieglitz, Caleb E. Finch, M. Linda Sutherland, James D. Sutherland, Helena C. Chui, Margaret Gatz, Wendy J. Mack, Hillard S. Kaplan, Andrei Irimia

**Affiliations:** 1 Alfred E. Mann Department of Biomedical Engineering, Viterbi School of Engineering, University of Southern California, Los Angeles, United States; 2 Ethel Percy Andrus Gerontology Center, Leonard Davis School of Gerontology, University of Southern California, Los Angeles, United States; 3 Department of Quantitative and Computational Biology, Dana and David Dornsife College of Letters, Arts and Sciences, University of Southern California, Los Angeles, United States; 4 Department of Radiology, Keck School of Medicine, University of Southern California, Los Angeles, United States; 5 School of Medicine, University of Illinois at Chicago, Chicago, United States; 6 Department of Physical Medicine and Rehabilitation, Harvard Medical School, Cambridge, United States; 7 Brain and Creativity Institute, University of Southern California, Los Angeles, United States; 8 Economic Science Institute, Argyros School of Business and Economics, Chapman University, Orange, United States; 9 Department of Neurosurgery, Stanford University, Stanford, United States; 10 Institute of Biomedical Research, Universidad Mayor de San Simon, Cochabamba, Bolivia; 11 Tsimane Health and Life History Project, San Borja, Bolivia; 12 Department of Anthropology, University of New Mexico, Albuquerque, United States; 13 School of Collective Intelligence, Université Mohammed VI Polytechnique, Marrakesh, Morocco; 14 Saint Luke’s Mid America Heart Institute, University of Missouri–Kansas City, Kansas City, United States; 15 Center for Evolution and Medicine, School of Human Evolution and Social Change, Arizona State University, Tempe, United States; 16 Institute of Human Origins, Arizona State University, Tempe, United States; 17 Department of Anthropology, University of California, Santa Barbara, Santa Barbara, United States; 18 Department of Social and Behavioral Sciences, Toulouse School of Economics, Toulouse, France; 19 Department of Biological Sciences, Dana and David Dornsife College of Letters, Arts and Sciences, University of Southern California, Los Angeles, United States; 20 Department of Anthropology, Dana and David Dornsife College of Letters, Arts and Sciences, University of Southern California, Los Angeles, United States; 21 Department of Psychology, Dana and David Dornsife College of Letters, Arts and Sciences, University of Southern California, Los Angeles, United States; 22 MemorialCare Health Systems, Fountain Valley, United States; 23 Department of Neurology, Keck School of Medicine, University of Southern California, Los Angeles, United States; 24 Center for Economic and Social Research, Dana and David Dornsife College of Letters, Arts and Sciences, University of Southern California, Los Angeles, United States; 25 Department of Population and Public Health Sciences, Keck School of Medicine, University of Southern California, Los Angeles, United States; 26 Institute of Psychiatry, Psychology and Neuroscience, Department of Psychological Medicine, Kings College London, London, United Kingdom

**Keywords:** Brain volume, Computed tomography, Intracranial artery calcification, Morphometry

## Abstract

**Purpose:**

Intracranial internal carotid artery calcification (iICAC) is a form of intracranial arteriosclerosis and is associated with an elevated risk of stroke and dementia. However, iICAC’s relationship with brain atrophy remains poorly understood. We aimed to automatically quantify iICAC morphometric characteristics and evaluate their associations with regional brain volumes (BVs).

**Methods:**

We developed an automated approach to compute iICAC surface area and thickness from CT brain scans in a sample of physically active South American subsistence farmers (*n* = 1,232, age range: 40 years to 92 years, 48.1% female, 794 Tsimane and 438 Moseten). Linear regression models were used to assess associations between two iICAC features and regional BVs, adjusted for age, sex, population, and total intracranial volume.

**Results:**

Significant negative relationships were found between regional BVs and iICAC surface area, but not iICAC thickness. Frontal, parietal, temporal, and subcortical BVs exhibited significant negative associations with iICAC surface area (standardized $\beta$ range: -0.146 to -0.066, *p* ≤ 0.013), while the occipital BV did not (standardized ${\beta}_{left}$ = -0.035, *p* = 0.249; ${\beta}_{right}$ = 0.007, *p* = 0.810). Subcortical BVs demonstrated the strongest negative associations with iICAC surface area (standardized ${\beta}_{left}$ = -0.146, *p* < 0.001; ${\beta}_{right}$ = -0.139, *p* < 0.001).

**Conclusion:**

iICAC surface area—assumed to reflect arterial stiffness—shows a stronger relationship with regional BV loss than iICAC thickness—assumed to indicate arterial stenosis. The findings suggest that brain regions primarily supplied by the anterior circulation are more vulnerable to iICAC-related atrophy. Subcortical BVs showed the strongest negative associations with iICAC surface area, with region-specific analyses identifying significant effects in the putamen, thalamus, hippocampus, amygdala, pallidum, and ventral diencephalon, suggesting heightened vulnerability of deep gray-matter structures to iICAC-related atrophy.

**Supplementary Information:**

The online version contains supplementary material available at 10.1007/s00234-026-03918-9.

## Introduction

Intracranial internal carotid artery calcification (iICAC) is a form of intracranial arteriosclerosis at the most prevalent site of calcification among all intracranial arterial beds [1]. iICAC can be detected from the first decade of life and increases with age, as found by a Dutch Emergency Department Study of individuals aged 1 to 100 years (y) [2]. Several studies have demonstrated that iICAC is associated with cardiovascular risk factors and with elevated risk of stroke [3, 4]. Despite the growing recognition of iICAC as a significant factor in cerebrovascular health, its relationship with brain atrophy, an imaging finding in aging [5], is not well understood. An autopsy study in the USA found that the effects of arteriosclerosis on cognitive impairments act through cortical gray matter atrophy [6, 7]. Regional brain atrophy is a common age-related phenomenon, progressing at different rates across sexes [8, 9], with distinct spatial and temporal patterns that can help to assess risk of neurodegenerative diseases, including Alzheimer’s disease (AD) [10]. The Rotterdam Study found that intracranial arteriosclerosis increases the risk of dementia [11]. Studying the relationship between brain atrophy and iICAC could therefore offer important insights into how vascular calcification may contribute to normative aging processes as well as to dementia-related neurodegeneration.

Computed tomography (CT) brain scans can detect arterial calcifications and are therefore used clinically to map iICAC. The traditional CT-based measurement of iICAC, however, is limited to volumetric assessment, which provides insufficient information on iICAC severity [1–4]. A more comprehensive understanding of iICAC requires a broader range of measurements to quantify the complex characteristics of calcification. The surface area *S* of iICAC, for instance, has potential to serve as a possible marker of arterial stiffness. As the vascular calcification process progresses, the surface area of the calcified region expands, reflecting a reduction in the arterial walls’ ability to adapt to changes in blood flow [12]. The diminished arterial elasticity, corresponding to increased *S*, is a key factor that may result in impaired cerebral blood flow and that could contribute to brain atrophy [13, 14]. On the other hand, iICAC thickness *T* is a measure that correlates with the degree of arterial blockage (i.e., stenosis). As *T* increases, the narrowing of the arterial lumen may reduce blood flow, leading to chronic ischemia and subsequent brain atrophy [15].

We present an automated, non-invasive method to directly calculate *S*, and *T* using the CT scans of 1,232 participants from two indigenous South American populations. The Tsimane and Moseten are populations in the Bolivian Amazon with distinct lifestyles compared to industrialized societies. The Tsimane (population ~ 17,000) follow a traditional subsistence lifestyle, while the neighboring population, the Moseten (population ~ 3,000), are more acculturated with greater access to modern amenities such as electricity, sanitation, medical services, and market foods [16–19]. Both groups maintain high physical activity levels and have fewer dementia risk factors (e.g., type 2 diabetes, cardiovascular disease, smoking) compared to industrialized populations [18, 20, 21]. These factors were individually associated with vascular calcification and brain atrophy in prior studies [22–26]. Because of fewer industrialization-related risk factors in the Tsimane and Moseten, studying iICAC and brain atrophy in these two populations reduces potential confounding and allows examination of the relationship between vascular calcification and brain atrophy in a context that more closely reflects their fundamental biological mechanisms. Beyond individual risk factors, prior work has also shown cross-population differences in iICAC prevalence between industrialized and non-industrialized groups: iICAC was present in 79% of Tsimane and Moseten adults [27], compared to 16.9% in a European population-based cohort [28]. These differences further motivate the study of iICAC in Indigenous populations, where lower exposure to industrialized lifestyle factors allows clearer investigation of fundamental vascular and neurodegenerative processes.

Previous studies commonly relied on manual assessment of CT to measure the morphological features of iICAC [29]. Some studies have utilized post-mortem brain autopsy [30], with inability to meaningfully correlate findings to pre-mortem measures. Some investigations used semi-quantitative grading schemes, assigning discrete categories to measures *T* [31–33]. This study provides an automated approach for iICAC quantification, reducing manual scoring effort and enabling continuous measurement of thickness based on 3D surface modeling. We then assess how *S* and *T* contribute to regional brain atrophy and compare the relative importance of these measures as correlates of brain volumes (BVs). We suggest that *S* reflects reduced arterial wall elasticity, the diminished ability of the intracranial internal carotid artery (iICA) to expand and contract in relation to arterial pulse waves, whereas *T* reflects stenotic restrictions on blood flow. We examine the relationships between *S* and *T* with regional BVs (i.e., frontal, parietal, occipital, temporal lobes, and subcortical regions) using multiple linear regression while controlling for age, sex, population, and total intracranial volume. Different brain regions vary in their vascular supply: the occipital lobe is primarily supplied by the posterior cerebral artery (PCA), whereas the other lobes are mainly perfused by the anterior (ACA) and middle cerebral arteries (MCA), both of which originate from the iICA [34–36]. Since iICAC reflects calcification in the internal carotid artery, we hypothesize that its association with brain atrophy is stronger in regions primarily supplied by the ACA or MCA, and weaker or negligible in regions predominantly supplied by the PCA.

## Materials and methods

### Participants

This study included 1,232 participants, comprising 794 Tsimane and 438 Moseten (48.1% female). The Tsimane are an indigenous population of forager-horticulturalists in lowland Bolivia, primarily depending on subsistence farming, hunting, fishing and gathering [16]. The Moseten, a neighboring population with similar genetic and ethnolinguistic background as the Tsimane, live as commercial farmers [17]. Detailed demographic information, including age and sex ratios for each population, are listed in Table S1 (Online Resource) and published elsewhere [8, 9]. For the Tsimane, ages range from 40 y to 92 y, with a mean of 59.9 y (SD = 10.3 y). For the Moseten, ages range from 40 y to 85 y, with a mean of 55.9 y (SD = 10.3 y). The proportions of women and men are similar across both populations.

### Imaging

CT scans of the brain were acquired for both Tsimane and Moseten participants between 2015 and 2018. A 16-detector row scanner (General Electric BrightSpeed, Milwaukee, WI) was used in the clockwise helical mode with a standard convolutional kernel. Reconstructions were performed with a matrix size of 512 × 512, generating two datasets: one at 1.25 mm resolution (*N* = 31) and another at 0.625 mm resolution (*N* = 1201). The small subset of participants scanned at 1.25 mm resolution did not correspond to a specific population, age range, or sex group. Additional scanning parameters included: kilovoltage peak = 120 kV; data collection diameter = 25 cm; mean exposure time = 1.417 s; X-ray tube current = 140 mA; focal spot = 0.7 mm [21].

#### iICAC segmentation

**Fig. 1:**
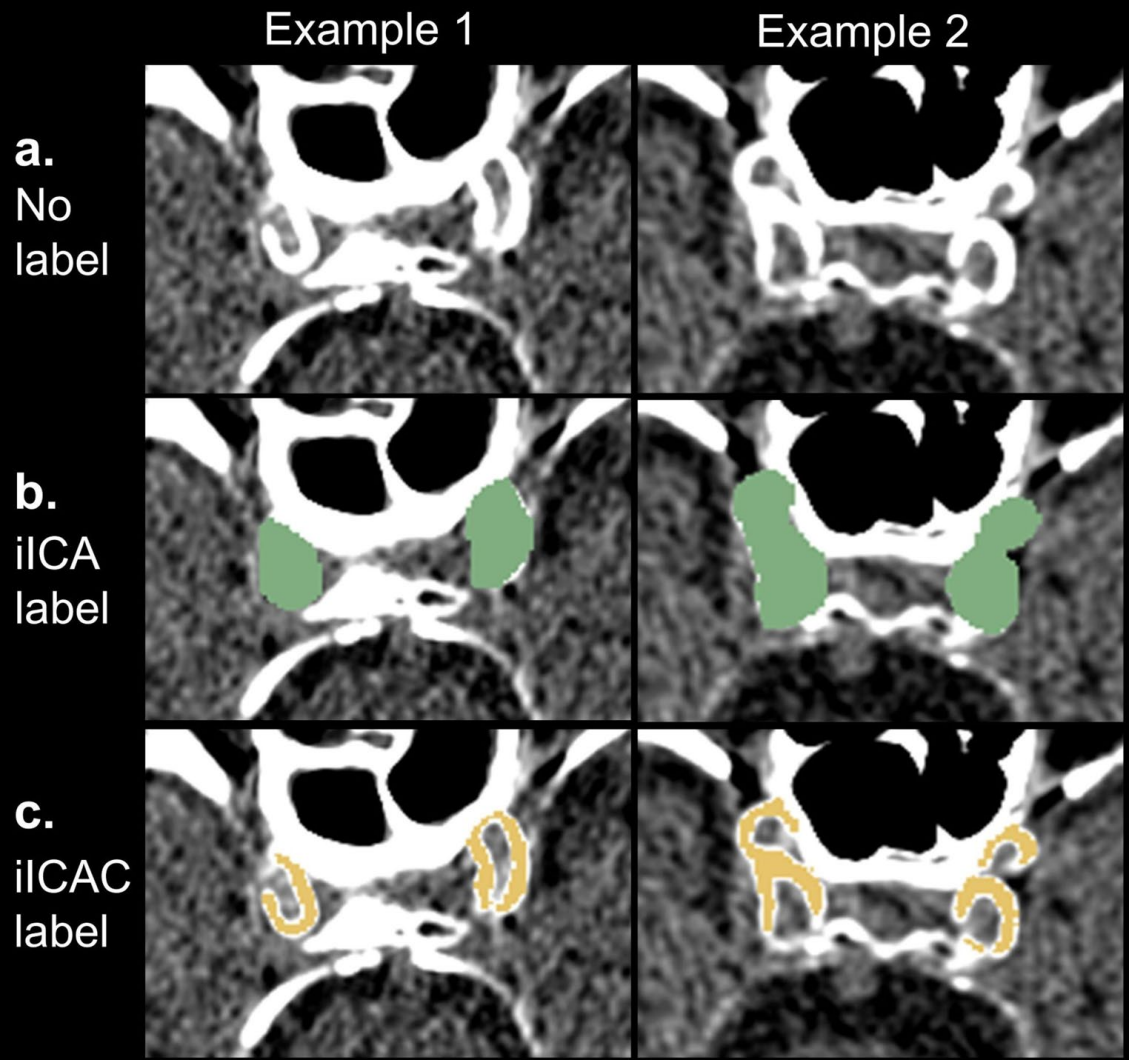
Two-step iICAC segmentation. **a** presents two example participants’ original CT scans in the axial view. **b** presents the intracranial internal carotid artery (iICA) voxels labeled in green by specialists in neuroimaging (step 1). **c** displays iICAC voxels (yellow) after thresholding between 130–500 HU (step 2)

Segmentation of iICAC from CT was performed in two steps (Fig. 1). First, three coders were trained and supervised by physician-scientist Giuseppe Barisano (Department of Neurosurgery, Stanford University) to manually labeled voxels corresponding to the iICA on the CT scans using ImageJ [37]. The iICA label covered the entire arterial cross-section, including both the vessel wall and the luminal space. Next, calcifications within the iICA were identified by thresholding CT attenuation values. Voxels with attenuation values between 130 and 500 Hounsfield units (HU) were considered calcified. The lower threshold value of 130 HU is commonly used in the calcium scoring method such as the Agatston score [38, 39]. The upper threshold value of 500 HU was applied to distinguish the vascular calcification from the bone close to the iICA. The iICAC volumetric measurement based on the manually labeled artery regions was validated by three independent raters, with an estimated intraclass correlation coefficient (ICC) of 0.97 (95% confidence interval: 0.91–0.99, *p* < 0.001), demonstrating excellent reliability and reproducibility.

### Reproducibility of iICAC segmentation across CT spatial resolutions

CT scans were acquired at two spatial resolutions (0.625 mm and 1.25 mm). To assess the reproducibility of iICAC segmentation across resolutions, we conducted a down-sampling analysis. CT scans originally acquired at 0.625 mm resolution (*N* = 1201), along with their manually labeled iICA lumen masks, were down-sampled to 1.25 mm resolution. The full iICAC segmentation pipeline was repeated on the down-sampled data, identifying calcified voxels within the lumen mask using the same attenuation threshold (130 HU–500 HU). Reproducibility between original and down-sampled iICAC volumes was evaluated using Pearson correlation, ICC, and mean absolute volume difference. CT scans at different spatial resolutions were processed using identical segmentation procedures without harmonization.

### iICAC *S* and *T* calculation

**Fig. 2:**
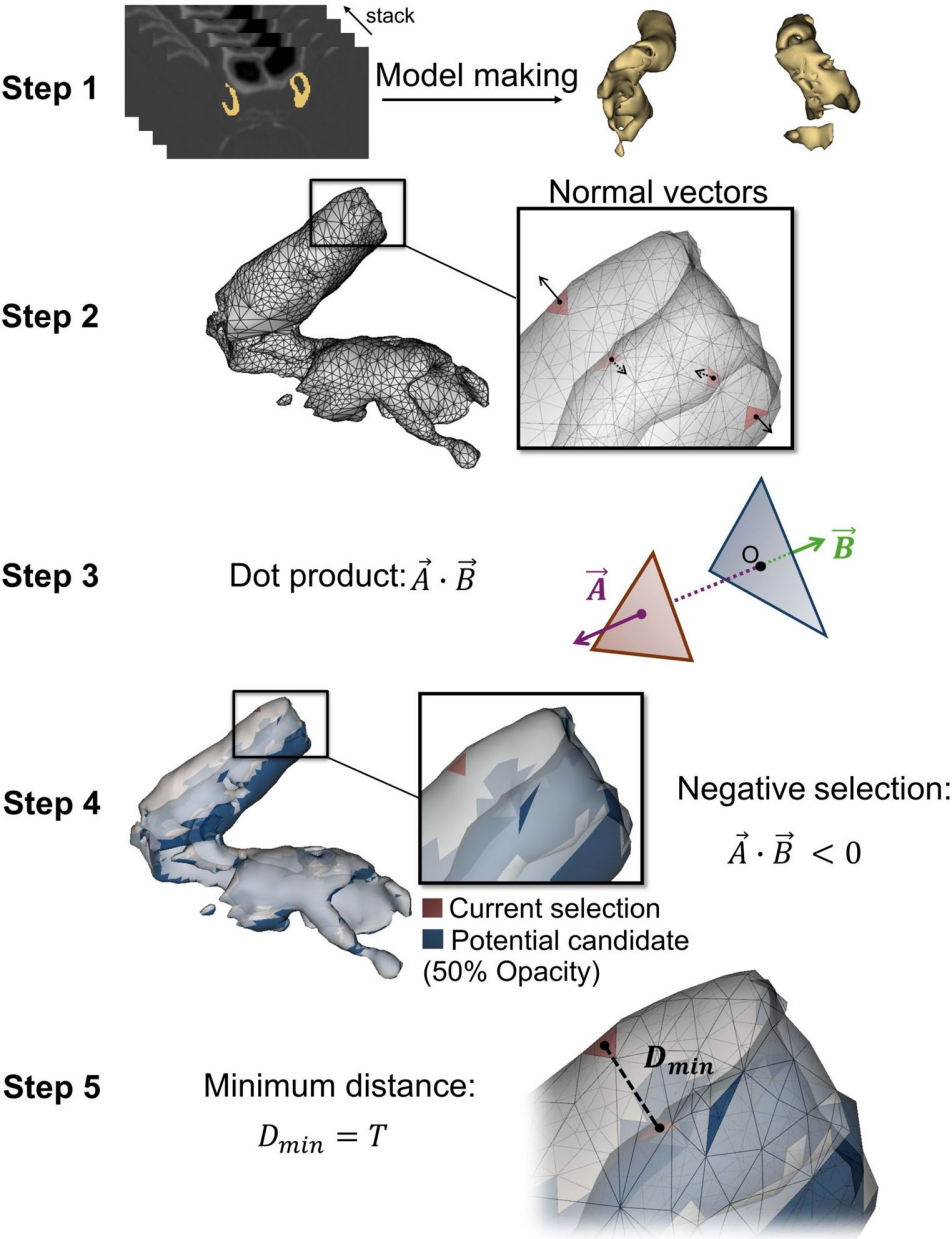
iICAC *S* and *T* calculation. **Step 1**: the iICAC label map of an example participant was converted to a 3D surface model. **Step 2**: The example participant’s right iICAC surface model is shown in detail. *S* was calculated by summing all the triangle meshes’ areas over the iICAC surface model. The normal vectors for random triangle meshes are displayed. **Step 3**: The dot product of a chosen triangle mesh’s normal vector ($\overrightarrow{A}$) and another triangle mesh’s normal vector ($\overrightarrow{B}$) was calculated. **Step 4**: Only the triangle meshes with negative dot products were selected as potential candidates to match the currently selected one. Potential candidates were on the other side (opposing surface) of the iICAC surface model. **Step 5**: The best candidate was chosen as the candidate with shortest distance to the currently selected triangle mesh. The distance between the centers of the selected triangle mesh and of the candidate mesh was seen as the *T* value at this point

A new pipeline was developed to calculate *S* and *T* at different points on the calcification surface for each participant. Figure 2 illustrates the five steps involved in the iICAC *S* and *T* calculation. First, for each participant, a 3D surface model of iICAC was constructed based on the iICAC label mask (Fig. 2, Step 1). 3D Slicer (www.slicer.org) [40] was used to construct a surface model representing the geometric structure of iICAC with a smoothing factor of zero. The iICAC surface model is composed of triangle meshes. *S* was measured via Visualization Toolkit (VTK) by summing all the triangle meshes’ areas over the iICAC surface model [41]. Second, to calculate *T*, normal vectors for all triangle meshes were obtained using VTK (Fig. 2, Step 2). Third, for each triangle mesh on the surface model, dot products were computed between (i) the currently selected triangle mesh’s normal vector ($\overrightarrow{A}$) and (ii) all other triangle meshes’ normal vectors ($\overrightarrow{B}$) (Fig. 2, Step 3). Fourth, $\overrightarrow{B}$ vectors with negative dot products ($\overrightarrow{A}\cdot\overrightarrow{B}<0$) were selected as normal vectors for potential candidates to pair with $\overrightarrow{A}$. This is because the negative sign of $\overrightarrow{A}\cdot\overrightarrow{B}$ indicates that the angle between $\overrightarrow{A}$ and $\overrightarrow{B}$ is over 90 degrees; thus, the two corresponding triangle meshes are on opposite sides of the surface (Fig. 2, Step 4). On the iICAC surface model, when two triangle meshes were not on the same side, the distance between them was likely to represent *T* at that point. Fifth, the distances between the current triangle mesh and all its potential pairing candidates were calculated (Fig. 2, Step 5). The final value of *T* on the current triangle mesh was identified as the shortest distance from its center point to the center point of candidate triangle meshes.

Following the fifth step, each *T* value measured over the iICAC surface model was weighted based on the radial direction of the iICA. Specifically, for each triangle mesh on the iICAC surface model, five nearest mesh triangles were selected on the iICA model and the angle between the normal vectors of each pair of mesh triangles was computed. If the average angle associated with the five triangles approached 90 degrees, a weight near zero was assigned. In contrast, if the average angle was closer to 0 degrees or 180 degrees, a higher weight, approaching 1, was assigned. This directional correction ensured that we only considered *T* values measured across the artery’s cross-sectional plane. Finally, the 97th percentile of all values of *T* over the surface model was selected as the representative parameter of *T* for each participant. This percentile was chosen because it effectively captures extreme iICAC thickness while remaining robust to local surface irregularities, providing a stable and clinically meaningful measure.

### Brain segmentation

The intracranial space was segmented using a probabilistic classification algorithm based on CT [42]. Before brain parcellation, each CT scan was converted to a high resolution T1-weighted MRI scan using the publicly available artificial intelligence tool SynthSR [43]. FreeSurfer was utilized to segment the cortical gray matter and to further parcellate it into 148 cortical regions and 14 subcortical structures (162 structures in total) based on the Destrieux parcellation scheme [44, 45]. Finally, the parcellated gray matter regions were mapped to 5 main brain regions in each hemisphere (10 total) – frontal, parietal, occipital, and temporal lobes, as well as subcortical regions [46]. Here, subcortical regions include the caudate nucleus, putamen, thalamus, hippocampus, amygdala, pallidum, and ventral diencephalon.

### Validation of CT-derived Synthetic MRI volumetrics

To evaluate the accuracy of CT-derived synthetic MRI brain parcellation volumes, we performed a validation using two independent datasets containing paired CT and T1-weighted MRI scans. For each participant, CT scans were converted to synthetic MRI using SynthSR and then processed through the same FreeSurfer parcellation pipeline described above to obtain 162 cortical and subcortical regional volumes.

The first dataset, the Irimia dataset [8], contains 13 participants with paired non-contrast CT and MRI scans. CT scans were acquired using a Toshiba Aquilion ONE scanner (120 kV, 140 mA, helical acquisition, FC68 convolution kernel; voxel size 0.46 mm × 0.46 mm × 0.60 mm). MRI scans were obtained on a 3 T Siemens Prisma system using a magnetization-prepared rapid gradient echo (MPRAGE) sequence (TR = 1950 ms, TE = 3 ms, TI = 900 ms, flip angle = 9°, voxel size = 1 mm × 1 mm × 1 mm).

The second dataset, SynthRAD2023 [47], is a publicly available benchmark from which we selected 59 paired non-contrast CT and T1-weighted MRI scans, all acquired at the University Medical Center Utrecht. CT scans were acquired on Philips Big Bore or Brilliance Big Bore scanners (120 kV, 234 mA–350 mA, 400 mAs–450 mAs, exposure time 1143ms–1712 ms, pixel spacing 0.57 mm–1.17 mm, slice thickness 1 mm–2 mm), and MRI scans were acquired on Philips Ingenia or Achieva dStream systems using a 3D spoiled T1-weighted gradient-echo sequence (flip angle = 8°, TE 3.48–4.06 ms, TR 7.63 ms–8.67 ms, pixel spacing 0.22 mm–0.96 mm).

For both datasets, participant-wise weighted absolute percent differences between synthetic MRI-derived and real MRI-derived regional volumes were calculated. Each region’s percentage difference was weighted by its proportion of total brain volume for the participant. Weighted differences were then averaged across participants to summarize volumetric discrepancies for each dataset.

### Regression analysis between iICAC measurements and regional BV

For each regional BV, three linear regression models were employed to examine their relationships with *S* and *T*. We evaluated collinearity across all independent variables by calculating the variance inflation factor (VIF). For model 1, the linear regression model of regional BV included *S* as a predictor, controlling for age, sex, population, and total intracranial volume (TICV). Model 2 used *T* as a predictor with the same control variables as model 1. Model 3 utilized both *S* and *T* as multiple predictors, again with the same control variables. Each dependent and independent variable was modeled as a standardized unit via *z*-scoring (except categorical variables of sex and population). The regression estimates reflect the mean difference of regional BV standard deviation (SD) units per 1 SD difference in the predictor. To control for multiple comparisons of regression estimates across brain regions, the Benjamini & Hochberg procedure was applied to control the false discovery rate [48].

## Results

### iICAC measurements

**Fig. 3:**
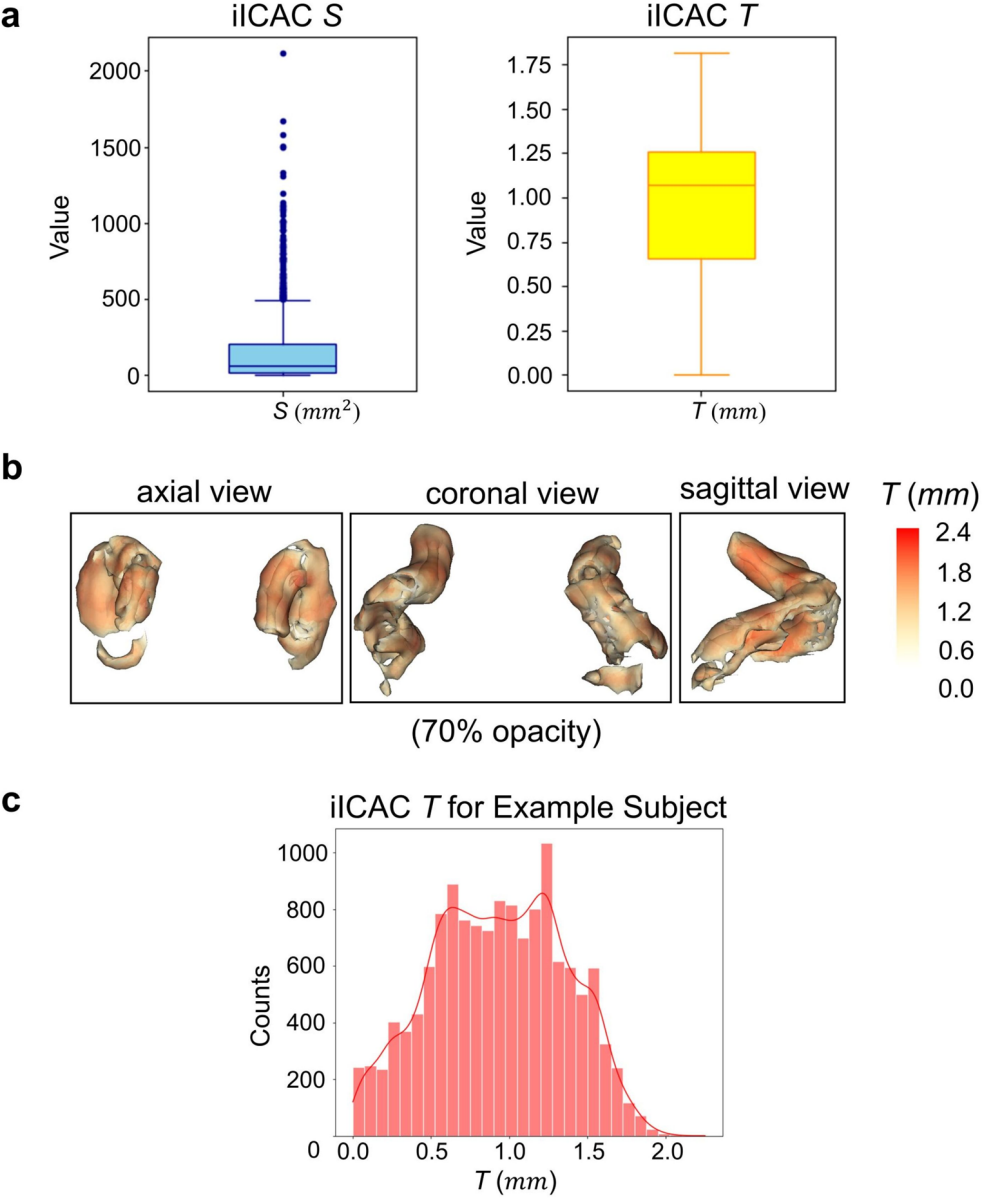
Distribution of *S* and *T* across participants (**a**) and one participant’s calculated *T* on different points of its iICAC surface model (**b**). **b** is the iICAC surface model (axial, coronal, and sagittal view) of one participant, where *T* on each point is represented by the varied color (darker red = thicker iICAC). The opacity of the model is reduced to 70% for clearer observation of its internal structure. **c** is the histogram of *T* values located on all points over this participant’s iICAC surface model

Table 1 presents the estimates of iICAC *S*, *T*, and their distribution by age. Both *S* and *T* significantly deviate from a normal distribution (*p* < 0.001) according to the Shapiro–Wilk test (Fig. 3A) [49]. The median of *S* is 62.75 mm^2^ with an interquartile range (IQR) of 193.35 mm^2^. For *T*, the median is 1.07 mm with an IQR of 0.60 mm. *S* and *T* are strongly correlated with each other (Spearman’s correlation = 0.862, *p* < 0.0001) [50]. Only 35 of 1,232 participants (2.8%) had no iICAC. 5.8% of participants exhibit *T* that is 1.50 mm or greater, consistent with a previous study in the Netherlands where approximately 5% of local participants had intimal iICAC defined as *T*$\ge$1.50 mm [30]. Age is associated positively with both *S* and *T* using Spearman correlation. As shown across decade-based age groups in Table 1, all participants aged 70 y and above exhibited iICAC, whereas 74% (26 of 35) of those without iICAC were in the youngest age group (40 y–49 y). There are no statistically significant population differences in *S* or *T*, with median *S* = 57.48 mm^2^ (IQR = 190.37 mm^2^), median *T* = 1.09 mm (IQR = 0.66 mm) in Moseten population and median *S* = 68.06 mm^2^ (IQR = 191.91 mm^2^), median *T* = 1.06 mm (IQR = 0.57 mm) in Tsimane population.

**Table 1:** iICAC *S* and *T* measurements distributed by age

iICAC measure	Median	IQR	Difference of measures between Tsimane & Moseten (U-test)			Correlation with age		
*S* (mm^2^)	62.75	193.35	0.0435 ($\:p=0.13$)			0.64 ($\:p<0.001$)		
*T* (mm)	1.07	0.60	0.0116 ($\:p=0.68$)			0.55 ($\:p<0.001$)		
			*S* (mm^2^)			*T* (mm)		
Age group	Size	Females: Males	Minimum	Median	IQR	Minimum	Median	IQR
40–49	305	1:0.87	0.00	13.99	38.25	0.00	0.63	0.77
50–59	394	1:1.20	0.00	41.97	107.64	0.00	0.98	0.59
60–69	345	1:1.08	0.00	136.32	246.00	0.00	1.18	0.37
70–79	147	1:1.41	1.48	352.22	400.86	0.23	1.31	0.21
80+	41	1:0.78	58.04	475.98	517.82	0.93	1.36	0.26

Figure 3B visualizes the *T* calculation results for a single participant. At each point over the surface of a 3D iICAC model, a *T* value was obtained. After adjusting the 3D model opacity to reveal the internal structure of the iICAC, it becomes evident that the calculated *T* values are higher in regions where the iICAC is thicker. The overall distribution of the *T* values over all points on the surface of this participant’s iICAC model is displayed in Fig. 3C.

### Reproducibility of iICAC measurements across CT spatial resolutions

iICAC volumes derived from original (0.625 mm resolution) and down-sampled (1.25 mm resolution) CT scans demonstrated near-perfect agreement. The Pearson correlation between original and down-sampled iICAC volumes was 0.999 (*p* < 0.001), and the ICC [1, 2], which quantifies the absolute agreement between single measurements while accounting for both systematic and random differences, ranged from 0.998 to 0.999. The mean absolute difference between measurements was 3.15 mm³ (SD = 3.75 mm³), which is small relative to the mean iICAC volume (54.45 mm³). A scatter plot (Fig. S1) further illustrates the high reproducibility of iICAC segmentation, showing that iICAC volumes from CT scans at different resolutions align almost perfectly along the identity line.

### Validation of CT-derived synthetic MRI volumetrics

Because acquiring MRI scans from the Tsimane/Moseten cohort was not feasible, CT scans were acquired instead. Regional brain volumes were extracted from CT scans converted to synthetic MRI using SynthSR, followed by segmentation with FreeSurfer. Validation of this process used paired CT-MRI brain scans. In the Irimia dataset, which consists of 13 participants with paired CT and MRI scans, the participant-wise weighted percent difference between SynthSR-derived and MRI-derived FreeSurfer volumes (over 162 structures) was 9.7% in median (IQR 5.8%). In the SynthRAD2023 dataset, which includes 59 paired CT and MRI scans, the corresponding mean weighted percent difference was 3.5% in median (IQR 2.0%).

### Relationship of iICAC to regional BVs

**Fig. 4:**
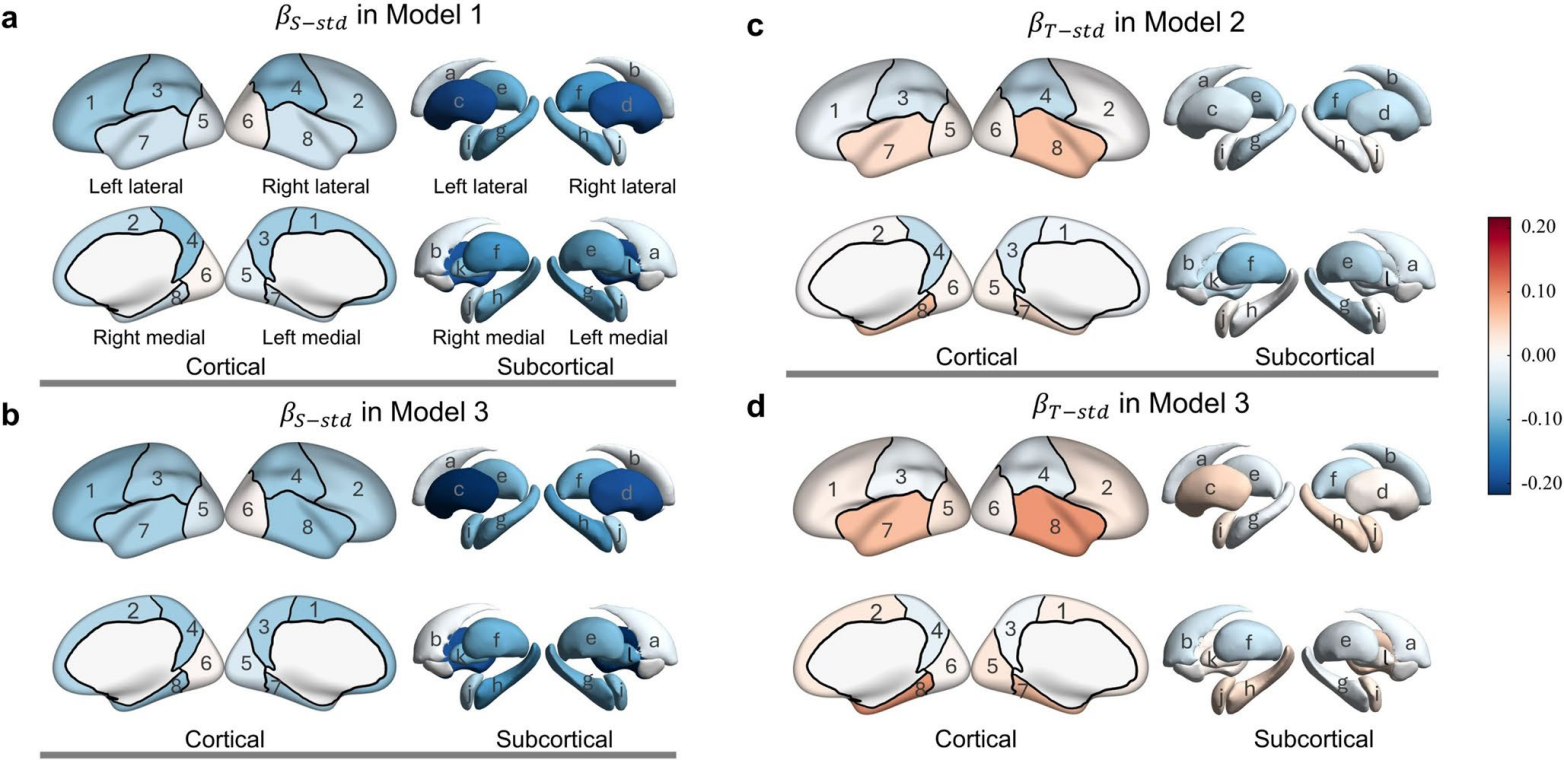
Standardized estimates distributed over 4 brain lobes in each hemisphere (8 total, each being labelled according to the index of Table 2) and 6 bilateral subcortical structures (12 total, each labelled according to the index of Table 3). **a** visualizes the standardized estimates of *S* from Model 1 where regional BVs are regressed only on *S*. **b** visualizes the standardized estimates of *S* from Model 3 where regional BVs are regressed on *S* and *T* simultaneously. **c** visualizes the standardized estimates of *T* from Model 2 where regional BVs are regressed only on *T*. **d** visualizes the standardized estimates of *T* from Model 3 where regional BVs are regressed on *S* and *T* simultaneously. Data were analyzed by linear regression, adjusted for age, sex, population, and total intracranial volume, after false discovery rate correction (Note: ${\beta}_{X-std}\equiv{\beta}^{\star}_{X}$)

For each regional BV, three linear regression models were used to examine the relationships of BV with iICAC measurements of *S* and *T*. VIFs of all variables were verified to be below 5, indicating no significant collinearity among independent variables. Here, the brain was segmented into 5 regions in each hemisphere (total of 10 BV regions), including the frontal, parietal, occipital, and temporal lobes, as well as subcortical regions. The subcortical regions included 14 structures, 7 in each hemisphere: the caudate nucleus, putamen, thalamus, hippocampus, amygdala, pallidum, and ventral diencephalon. We expected stronger associations of iICAC measurements to brain atrophy in regions supplied by the ACA or MCA, and weaker or no associations in regions supplied by the PCA. Regression Model 1 and 2 reveal how *S* or *T* independently contributes to these 10 regional BVs. Model 3 compares the relative importance of *S* and *T* in their contribution to regional BVs. Table 2 lists the regression estimates from Models 1, 2 and 3 regressed on 10 regional BVs. In addition, we applied these three models to 162 regional BVs (148 cortical, 14 subcortical) further parcellated based on the Destrieux atlas. Table 3 displays the regression results for 14 subcortical structures from Models 1, 2, and 3 regressed on 162 regional BVs. Figure 4A and B visualize the estimates of S over brain lobes and further-parcellated subcortical structures from Model 1 and 3, respectively. Figure 4C and D visualize the estimates of *T* over brain lobes and further-parcellated subcortical structures from Model 2 and 3, respectively. The regression results for 148 cortical regions from Model 1, 2, and 3 are shown in Table S2, S3, and S4 (Online Resource), respectively, and visualized in Fig. S2 (Online Resource). The false discovery rate correction was applied to all regression models [48].

**Table 2:** Linear regression model 1, 2, and 3 where 10 regional BVs are regressed on *S*, on *T*, and on *S* as well as *T* simultaneously, controlling for age, sex, population, and total intracranial volume, after false discovery rate correction. model specifications use Wilkinson notation, e.g., BV ~ S indicates that S predicts BV

				Model 1: BV ~ S			Model 2: BV ~ T		
Index		Hemi	Region	${\beta}_{S} ({\beta}^{\star}_{S})$	SE	*p*	${\beta}_{T} ({\beta}^{\star}_{T})$	SE	*p*
1		L	frontal	**−2.17 (−0.079)**	0.581	**< 0.001**	−213.4 (−0.013)	332.2	0.729
2		R	frontal	**−1.54 (−0.055)**	0.594	** 0.022**	41.3 (0.002)	338.3	0.903
3		L	parietal	**−1.37 (−0.077)**	0.447	** 0.006**	−368.1 (−0.035)	254.7	0.297
4		R	parietal	**−1.65 (−0.089)**	0.454	** 0.001**	−597.5 (−0.055)	258.7	0.059
5		L	occipital	−0.39 (−0.023)	0.436	0.473	161.9 (0.016)	247.6	0.729
6		R	occipital	0.18 (0.010)	0.484	0.827	107.3 (0.010)	274.9	0.813
7		L	temporal	−0.75 (−0.045)	0.417	0.125	401.2 (0.040)	237.1	0.212
8		R	temporal	−0.78 (−0.044)	0.454	0.135	** 669.8 (0.064)**	257.5	**0.044**
9		L	subcortical	**−1.30 (−0.145)**	0.200	**< 0.001**	** −285.9 (−0.054)**	115.3	**0.047**
10		R	subcortical	**−1.28 (−0.142)**	0.199	**< 0.001**	** −321.9 (−0.061)**	114.6	**0.044**
				Model 3: BV ~ *S* + *T*					
Index		Hemi	Region	${\beta}_{S} ({\beta}^{\star}_{S})$	SE	*p*	${\beta}_{T} ({\beta}^{\star}_{T})$	SE	*p*
1		L	frontal	**−2.39 (−0.086)**	0.631	** 0.001**	314.5 (0.019)	358.6	0.592
2		R	frontal	**−1.85 (−0.066)**	0.644	** 0.010**	450.1 (0.027)	366.1	0.592
3		L	parietal	**−1.32 (−0.074)**	0.485	** 0.013**	−75.8 (−0.007)	275.8	0.851
4		R	parietal	**−1.46 (−0.079)**	0.493	** 0.009**	−274.9 (−0.025)	279.9	0.592
5		L	occipital	−0.59 (−0.035)	0.473	0.249	292.2 (0.030)	268.7	0.592
6		R	occipital	0.13 (0.007)	0.525	0.810	79.4(0.007)	298.6	0.851
7		L	temporal	**−1.21 (−0.072)**	0.452	** 0.013**	** 669.0 (0.067)**	256.7	**0.043**
8		R	temporal	**−1.46 (−0.082)**	0.490	** 0.009**	** 992.0 (0.095)**	278.6	**0.005**
9		L	subcortical	**−1.30 (−0.146)**	0.217	**< 0.001**	1.4 (< 0.001)	123.3	0.991
10		R	subcortical	**−1.25 (−0.139)**	0.216	**< 0.001**	−45.9 (−0.009)	122.7	0.851

**Table 3:** Regression results for 14 subcortical regions (ventral DC refers to the ventral diencephalon) from model 1, 2, and 3 where totally 162 regional BVs (parcellated based on Destrieux atlas) are regressed on *S*, on *T*, and on *S* as well as *T* simultaneously, controlling for age, sex, population, and total intracranial volume, after false discovery rate correction. model specifications use Wilkinson notation, e.g., BV ~ S indicates that S predicts BV

			Model 1: BV ~ S			Model 2: BV ~ T		
Index	Hemi	Region	${\beta}^{\star}_{S}$	SE	*p*	${\beta}^{\star}_{T}$	SE	*p*
a	L	Caudate	−0.031	0.029	0.467	−0.028	0.028	0.629
b	R	Caudate	−0.026	0.028	0.556	−0.046	0.027	0.334
c	L	Putamen	**−0.198**	0.029	**< 0.001**	−0.035	0.028	0.559
d	R	Putamen	**−0.187**	0.029	**< 0.001**	−0.058	0.029	0.231
e	L	Thalamus	**−0.109**	0.027	** 0.001**	−0.063	0.026	0.166
f	R	Thalamus	**−0.129**	0.026	**< 0.001**	**−0.092**	0.025	**0.013**
g	L	Hippocampus	**−0.112**	0.026	**< 0.001**	−0.054	0.025	0.215
h	R	Hippocampus	**−0.108**	0.026	** 0.001**	−0.006	0.025	0.952
i	L	Amygdala	**−0.083**	0.028	** 0.020**	−0.004	0.027	0.971
j	R	Amygdala	−0.053	0.028	0.181	0.013	0.027	0.814
k	L	Pallidum	**−0.111**	0.027	** 0.001**	−0.030	0.026	0.572
l	R	Pallidum	**−0.096**	0.028	** 0.004**	−0.019	0.027	0.733
m	L	Ventral DC	**−0.129**	0.026	**< 0.001**	**−0.110**	0.025	**0.003**
n	R	Ventral DC	**−0.134**	0.025	**< 0.001**	**−0.077**	0.025	**0.037**
			Model 3: BV ~ *S* + *T*					
Index	Hemi	Region	${\beta}^{\star}_{S}$	SE	*p*	${\beta}^{\star}_{T}$	SE	*p*
a	L	Caudate	−0.023	0.031	0.677	−0.020	0.030	0.806
b	R	Caudate	−0.009	0.030	0.894	−0.043	0.029	0.505
c	L	Putamen	**−0.217**	0.031	**< 0.001**	0.046	0.030	0.505
d	R	Putamen	**−0.192**	0.032	**< 0.001**	0.014	0.031	0.863
e	L	Thalamus	**−0.098**	0.030	** 0.008**	−0.026	0.028	0.659
f	R	Thalamus	**−0.107**	0.029	** 0.002**	−0.052	0.028	0.390
g	L	Hippocampus	**−0.106**	0.029	** 0.003**	−0.014	0.027	0.863
h	R	Hippocampus	**−0.124**	0.028	**< 0.001**	0.040	0.027	0.505
i	L	Amygdala	**−0.096**	0.030	** 0.012**	0.031	0.029	0.634
j	R	Amygdala	−0.069	0.030	0.094	0.039	0.029	0.545
k	L	Pallidum	**−0.117**	0.030	** 0.001**	0.014	0.028	0.863
l	R	Pallidum	**−0.104**	0.030	** 0.006**	0.020	0.029	0.788
m	L	Ventral DC	**−0.100**	0.029	** 0.005**	−0.072	0.027	0.255
n	R	Ventral DC	**−0.121**	0.028	**< 0.001**	−0.032	0.027	0.606

In Model 1, where 10 regional BVs are regressed on *S* (Table 2), we find that after controlling for age, sex, population, and TICV, 60% (6 of 10) of brain regions are significantly and negatively associated with *S*. These regions include the frontal and parietal lobes, as well as the subcortical structures in each hemisphere (see Fig. 4A for the standardized estimates visualized over brain regions). The standardized estimate ${\beta}^{\star}_{S}$ reflects the mean difference in SD units of regional BV associated with a one SD difference in *S*. This measure enables us to compare the magnitude of estimates between regions. Bilaterally, compared with other brain regions, the subcortical regions have the highest magnitudes of ${\beta}^{\star}_{S}$, displaying the strongest trend in BV loss associated with *S*. In the same model where 162 regional BVs are regressed on *S*, most subcortical structures’ volumes, including each hemisphere’s thalamus, hippocampus, putamen, pallidum, ventral diencephalon, as well as the left amygdala, are significantly and negatively associated with *S*, except the bilateral caudate nucleus. Table 3 shows that bilateral putamen volumes have the strongest negative association with *S* among all the 162 regions (${\beta}^{\star}_{S}$ visualized over subcortical structures in Fig. 4A).

Model 2 in Table 2 presents the comparable linear regression models of 10 regional BVs on *T*, again controlling for age, sex, population, and TICV (standardized estimates visualized over brain regions are shown in Fig. 4C). Compared with Model 1, where regional BVs are regressed on *S*, fewer regional BVs are significantly and negatively associated with *T*. Only the subcortical regions of both hemispheres have significantly smaller BVs with larger *T*. The subcortical ${\beta}^{\star}_{T}$s from Model 2 are lower in magnitude than the subcortical ${\beta}^{\star}_{S}$s from Model 1, indicating the weaker contribution of *T* to subcortical BV loss compared to *S*. In addition, there is one marginally significant positive ${\beta}_{T}$ in Model 2, indicating a potential positive relationship between *T* and the volume of the right temporal lobe. It is also noticeable that, in both Models 1 and 2, no significant relationship is found between either hemisphere’s occipital lobe volume and *S* or *T*. This result is anatomically expected, as the occipital lobe is perfused by the posterior circulation (which is not directly supplied by the iICA).

Model 3 in Table 2 compares the contribution of *S* and *T* to regional BV, where both *S* and *T* were simultaneously modeled as predictors controlling for age, sex, population, and TICV (see Fig. 4B and D for standardized estimates visualized over brain regions). Collinearity is not a concern in Model 3, with low VIFs(below 2.00) of *S* and *T*. The significant estimates of *S* (${\beta}_{S}$) are negative while those of *T* (${\beta}_{T}$) are positive. Moreover, 6 more (8 compared to 2) regional BVs exhibit significant associations with *S* than with *T*. Compared to Model 1 where regional BVs are regressed only on *S*, the volumes of both temporal lobes are significantly negatively associated with *S* in Model 3 with adjustment for *T*. Additionally, two significant negative ${\beta}_{T}$s related to both sides of the subcortical region in Model 2 (where regional BVs are regressed only on *T*) are non-significant in Model 3, with the ${\beta}_{T}$ related to the left subcortical region turning positive. The magnitudes of both subcortical ${\beta}^{\star}_{T}$s are substantially reduced from Model 2 to Model 3, while the magnitudes of both subcortical ${\beta}^{\star}_{S}$s remain similar. The statistical non-significance of ${\beta}^{\star}_{T}$ estimates with adjustment for *S*, as well as the substantial reduction in magnitude of regression coefficients suggest that the impact from *T* on regional BV is smaller than that from *S*. 

## Discussion

This study analyzed the relationship between iICAC and regional BV using a novel automated method to quantify iICAC *S* and *T* from CT images. Our findings identify a statistically significant association between *S* and regional BV loss. The subcortical BVs exhibit the strongest negative associations with *S* among all brain regions, suggesting their heightened vulnerability to atrophy related to increased *S*. Compared to *S*, *T* exhibits a relatively weaker association with regional brain atrophy, suggesting that arterial stiffness indicated by *S* may contribute more to brain atrophy compared to arterial stenosis indicated by *T*.

### iICAC-related change in BV

Model 1 (using *S* as a predictor of regional BVs) and Model 3 (using both *S* and *T* as predictors) revealed statistically significant negative relationships between *S* and regional BVs. Specifically, 90% (9 of 10) of regional BVs in Model 1 using only an *S* predictor exhibited negative ${\beta}_{S}$ estimates, with 6 reaching statistical significance. In Model 3 (using both *S* and *T* as predictors), the same 90% (9 of 10) ${\beta}_{S}$ estimates were negative, but with 8 being statistically significant. These patterns suggest a trend in which greater *S* is associated with smaller BV across multiple brain regions (i.e., frontal, parietal, temporal) primarily supplied by anterior and middle circulation but not posterior circulation. However, further longitudinal data are needed to establish a causal relationship and to evaluate the utility of *S* as a predictor of developing brain atrophy.

Bilaterally, subcortical BVs exhibit the strongest negative associations with *S* ($\left|{\beta}^{\star}_{S}\right|\ge$0.100) in Model 1 (where regional BVs are regressed on *S*) and in Model 3 (with both *S* and *T* as predictors). This suggests that the subcortical structures, including the putamen, thalamus, hippocampus, amygdala, pallidum, and ventral diencephalon, are more vulnerable to volume loss associated with increasing *S* than other regions. This heightened vulnerability may be explained by two anatomical factors. First, the lenticulostriate arteries (LSAs), which arise from the M1 segment of the MCA and supply subcortical structures, are small in diameter. This makes them more sensitive to alterations in cerebral blood flow [51]. Second, the LSAs originate from the M1 segment at right angles, amplifying the transmission of pulsatile stress [52]. Since the MCA originates from the iICA, these findings align with the hypothesis that iICAC, particularly when extensive in surface area, impairs perfusion to deep brain structures more severely than to cortical regions. Among all the subcortical structures, the putamen (one of the basal ganglia) exhibits the strongest negative association with *S* (Table 3, visualized in Fig. 4A and B), indicating the volume loss of the putamen is most possible with increased *S*. Atrophy of the putamen was found by a study in the Netherlands to be significantly correlated with cognitive decline in AD [53]. The negative association between *S* and the subcortical BV indicates that the atrophy of deeper brain regions may be more associated with iICAC calcification and warrants further investigation into the relationship between iICAC and neurodegenerative diseases related to this atrophy.

Although some regions (notably the right temporal lobe) showed modest positive β values for *T*, these effects were small and not statistically robust. Given that *T* represents only a localized maximal thickness measurement and is therefore more sensitive to noise, these isolated positive associations should be interpreted with caution. By contrast, *S* reflects the global calcification burden and consistently demonstrates stronger, more biologically plausible associations with regional brain volume. Future studies with larger cohorts will be needed to determine whether the localized temporal findings related to *T* are reproducible.

### Arterial stiffness compared with stenosis

There are two main mechanisms hypothesized to explain how changes in arterial properties impact blood flow and contribute to brain atrophy: arterial stiffness and arterial stenosis. Arterial stiffness reduces the artery’s ability to expand and contract with each heartbeat, leading to impaired cerebral blood flow regulation [54, 55]. This loss of elasticity increases pulsatile pressure, damages small vessels, and disrupts cerebral autoregulation, which may contribute to chronic hypoperfusion and brain atrophy [13, 14, 56, 57]. As iICAC increases, the expansion of *S* may cause reduced elasticity of iICA walls, which makes *S* a potentially representative parameter of arterial stiffness. In contrast, arterial stenosis is assumed to increase when *T* grows and narrows the artery lumen, restricting blood flow and reducing oxygen supply to the brain, potentially accelerating neurodegeneration and volume loss [15]. In our study, *S*, which is assumed to possibly indicate arterial stiffness, exhibited a significant negative relationship with regional BV. By contrast, *T*, assumed to represent arterial stenosis, did not. When *S* is modeled as an independent variable along with *T*, from Model 2 to Model 3, the standardized estimates of *T* (${\beta}^{\star}_{T}$) on subcortical BV become smaller in magnitude and change from negative to positive. This suggests that the surface area of calcification, which is assumed to indicate arterial stiffness, has a more profound association with brain atrophy compared to the thickness of calcification. Arterial stenosis, as assumed to be represented by *T*, appears to play very little role in brain atrophy in this population beyond arterial stiffness (which is assumed to be explained by *S*). These results provide valuable insights into the mechanisms by which arterial calcification influences cerebral blood flow and potentially causes brain atrophy, indicating the potential importance of preserving arterial elasticity in preventing vascular contributions to brain dysfunction. 

### Indirect effects of iICAC on brain regions

Brain structures are not directly fed by the iICA, but by the ACA, MCA, and PCA [34]. Among the 4 brain lobes, 3 of them are primarily fed by the ACA or MCA, while the occipital lobe is primarily fed by the PCA [35]. The ACA and MCA arise from the terminal bifurcation of the iICA, while the PCA does not [36]. This explains the finding that no significant relationship was found between the volume of occipital lobe and iICAC *S* or *T*.

In addition, downstream brain regions far from the iICA, such as frontal and parietal lobes, were more affected by *S* compared to the temporal lobe closer to the iICA. This may be because the expansion of iICAC S, which is assumed to increase arterial stiffness and reduce pulsatility, might still allow some blood to reach the lobes closer to the iICA but might allow less blood to reach downstream lobes farther from the iICA. The subcortical region supplied by the LSAs has the strongest negative association with *S*, which may be explained by several anatomical and hemodynamic features of LSAs. First, the LSAs arise perpendicularly from the M1 segment of the MCA [52]. When iICAC reduces the blood flow within parent arteries, the right-angle origin of LSAs may further impede perfusion to subcortical regions. Second, the LSAs are terminal arteries with no significant collateral circulation, rendering their territories particularly vulnerable to hypoperfusion [58]. Third, the small size of LSAs reduces perfusion to subcortical structures when cerebral blood flow is altered by iICAC from the origin arteries [51].

### Limitations

This study introduces a novel automated method for quantifying the complex morphological features of iICAC, including *S* and *T*, and provides valuable insights into the relationship between iICAC and regional brain atrophy. Nonetheless, several limitations should be acknowledged. First, while studying indigenous populations offers unique insights into iICAC and brain atrophy among groups with low coronary atherosclerosis living in non-industrialized settings, it also limits the generalizability of our findings. Future studies should compare these associations with results in other populations with different lifestyles, and should test the role of vascular risk factors for dementia that are presumably more frequent in more industrialized populations. Second, the cross-sectional nature of the study limits our ability to draw conclusions about causal relationships between iICAC expansion and brain atrophy. Longitudinal studies are needed to better understand the temporal progression of iICAC morphometry and its potential impacts on brain health over time. Third, the measurement of *T* was constrained by the spatial resolution of the CT scans. Utilizing a CT scanner with higher spatial resolution could improve the precision of *T* quantification, particularly for capturing finer morphological details. Fourth, while we focused on *S* as a proxy for arterial stiffness, more direct measurements of stiffness, such as pulse wave velocity or other mechanical properties, could provide a more precise understanding of how arterial stiffness influences brain structures. Future research should incorporate more direct stiffness assessments to enhance the accuracy and depth of findings regarding the impact of iICAC on brain atrophy and to validate *S* as a marker of arterial stiffness. Fifth, the *T* metric used in this study does not distinguish whether iICAC thickening occurs inward, narrowing the arterial lumen, or outward, expanding beyond the vessel wall. This limitation arises because the segmentation relies on a lumen mask including both the artery wall and the space inside the vessel. Future studies with accurate arterial wall labeling could enable a stricter measure of narrowness that separates inward versus outward growth of iICAC. Sixth, the CT-derived synthetic MRI volumes remain a limitation. Validation on independent datasets (Irimia and SynthRAD2023) showed participant-wise weighted percent differences below 12% compared to real MRI-derived volumes, indicating generally acceptable agreement but also highlighting residual discrepancies. Future work should also aim to improve brain segmentation pipelines to further enhance volumetric accuracy. It may also be important to note that conducting MRI in this population is not feasible, because there are no MRI facilities within a reasonable travel distance from the population; thus, CT remains the only available modality.

## Supplementary Information

Below is the link to the electronic supplementary material.

Supplementary Material 1 (PDF 789 KB)

## Data Availability

Individual-level data are stored in the Tsimane Health and Life History Project Data Repository, and are available through restricted access for ethical reasons. Tsimane Health and Life History Project’s highest priority is the safeguarding of human subjects and minimization of risk to study participants. The Tsimane Health and Life History Project adheres to the “CARE Principles for Indigenous Data Governance” (Collective Benefit, Authority to Control, Responsibility, and Ethics), which assure that the Tsimane (1) have sovereignty over how data are shared, (2) are the primary gatekeepers determining ethical use, (3) are actively engaged in the data generation, and (4) derive benefit from data generated and shared for use whenever possible. The Tsimane Health and Life History Project is also committed to the “FAIR Guiding Principles for scientific data management and stewardship” (Findable, Accessible, Interoperable, Reusable). Requests for individual-level data should take the form of an application that details the exact uses of the data and the research questions to be addressed, procedures that will be employed for data security and individual privacy, potential benefits to the study communities, and procedures for assessing and minimizing stigmatizing interpretations of the research results (see the following webpage for links to the data sharing policy and data request forms: https://tsimane.anth.ucsb.edu/data.html). Requests for individual-level data will require institutional IRB approval (even if exempt) and will be reviewed by an Advisory Council composed of Tsimane community leaders, community members, Bolivian scientists, and the Tsimane Health and Life History Project leadership. The study authors and the Tsimane Health and Life History Project leadership are committed to open science and are available to assist interested investigators in preparing data access requests.
