# Supplementary material for: Segmentation and morphometry of intracranial internal carotid artery calcification in relation to brain atrophy
Source: Neuroradiology. 2026 Mar 14;68(4):911–26. doi: 10.1007/s00234-026-03918-9 (PMC13139289; doi:10.1007/s00234-026-03918-9)
Supplement: Supplementary file 1 — Supplementary Material 1 (PDF 789 KB) [file 234_2026_3918_MOESM1_ESM.pdf]

Table S1 Participant demographics for two populations (standard deviation shorted as SD)

Repository	N	Min (age)	Max (age)	Mean (age)	SD (age)	Males:Females
Tsimane	794	40	92	59.9	10.3	1:0.94
Moseten	438	40	85	55.9	10.3	1:0.90

Table S2 Linear regression results for 148 cortical regions (G refers to gyrus and S refers to sulcus) via Model 1 where totally 162 regional BVs (parcellated based on Destrieux atlas) are regressed on iICAC S, controlling for age, sex, population, and total intracranial volume, after false discovery rate correction

		β_{S-std}		SE		*p*	
Index	Region	Lh	Rh	Lh	Rh	Lh	Rh
1	G_and_S_frontomargin	**-0.098**	**-0.093**	0.035	0.035	**0.024**	**0.039**
2	G_and_S_occipital_inf	-0.009	-0.017	0.034	0.033	0.875	0.786
3	G_and_S_paracentral	-0.069	-0.062	0.032	0.032	0.111	0.170
4	G_and_S_subcentral	**-0.128**	**-0.115**	0.031	0.032	**0.001**	**0.003**
5	G_and_S_transv_frontopol	**-0.115**	**-0.086**	0.034	0.033	**0.005**	**0.042**
6	G_and_S_cingul-Ant	-0.004	0.013	0.028	0.027	0.917	0.790
7	G_and_S_cingul-Mid-Ant	-0.006	0.019	0.031	0.030	0.900	0.717
8	G_and_S_cingul-Mid-Post	**-0.102**	-0.073	0.032	0.031	**0.009**	0.067
9	G_cingul-Post-dorsal	**-0.088**	-0.048	0.032	0.033	**0.029**	0.296
10	G_cingul-Post-ventral	-0.035	-0.042	0.035	0.036	0.504	0.421
11	G_cuneus	0.007	0.035	0.033	0.032	0.900	0.455
12	G_front_inf-Opercular	-0.084	**-0.096**	0.033	0.034	0.053	**0.025**
13	G_front_inf-Orbital	-0.014	-0.009	0.035	0.035	0.839	0.875
14	G_front_inf-Triangul	-0.061	-0.058	0.034	0.035	0.207	0.223
15	G_front_middle	-0.042	-0.026	0.029	0.030	0.316	0.591
16	G_front_sup	-0.039	-0.001	0.028	0.028	0.316	0.975
17	G_Ins_lg_and_S_cent_ins	-0.014	-0.020	0.031	0.031	0.798	0.716
18	G_insular_short	-0.039	-0.051	0.030	0.031	0.374	0.223
19	G_occipital_middle	-0.063	-0.044	0.034	0.034	0.182	0.374
20	G_occipital_sup	-0.075	-0.061	0.035	0.035	0.111	0.218
21	G_oc-temp_lat-fusifor	0.037	0.063	0.033	0.032	0.448	0.161
22	G_oc-temp_med-Lingual	0.004	0.034	0.033	0.031	0.928	0.452
23	G_oc-temp_med-Parahip	-0.010	0.023	0.033	0.033	0.875	0.691
24	G_orbital	-0.048	-0.037	0.029	0.029	0.226	0.379
25	G_pariet_inf-Angular	-0.081	-0.075	0.033	0.032	0.059	0.079
26	G_pariet_inf-Supramar	**-0.113**	**-0.118**	0.031	0.031	**0.002**	**0.002**
27	G_parietal_sup	-0.063	-0.050	0.033	0.032	0.170	0.270
28	G_postcentral	-0.059	**-0.082**	0.031	0.031	0.181	0.042
29	G_precentral	**-0.154**	**-0.117**	0.030	0.031	**<0.001**	**0.002**
30	G_precuneus	-0.019	-0.008	0.031	0.032	0.738	0.875
31	G_rectus	-0.043	**-0.094**	0.032	0.033	0.349	**0.024**
32	G_subcallosal	-0.021	-0.051	0.032	0.031	0.708	0.223
33	G_temp_sup-G_T_transv	**-0.093**	-0.044	0.032	0.033	0.021	0.352
34	G_temp_sup-Lateral	**-0.124**	**-0.120**	0.032	0.032	**0.001**	**0.002**
35	G_temp_sup-Plan_polar	0.007	-0.054	0.030	0.030	0.886	0.195
36	G_temp_sup-Plan_tempo	-0.063	-0.014	0.035	0.035	0.195	0.839
37	G_temporal_inf	0.003	0.016	0.033	0.033	0.955	0.798
38	G_temporal_middle	-0.024	-0.046	0.031	0.031	0.643	0.296
39	Lat_Fis-ant-Horizont	-0.012	-0.065	0.035	0.034	0.859	0.181
40	Lat_Fis-ant-Vertical	**0.094**	0.013	0.036	0.035	**0.039**	0.839
41	Lat_Fis-post	**-0.096**	-0.044	0.031	0.030	**0.015**	0.306
42	Pole_occipital	-0.047	-0.038	0.033	0.032	0.317	0.421
43	Pole_temporal	**-0.088**	-0.060	0.034	0.033	**0.040**	0.195
44	S_calcarine	-0.033	0.028	0.033	0.032	0.504	0.580
45	S_central	-0.006	0.008	0.032	0.033	0.904	0.886
46	S_cingul-Marginalis	-0.009	-0.045	0.034	0.033	0.875	0.342
47	S_circular_insula_ant	0.069	0.046	0.032	0.031	0.111	0.295
48	S_circular_insula_inf	0.013	0.035	0.027	0.028	0.798	0.391
49	S_circular_insula_sup	0.053	0.073	0.030	0.029	0.208	0.055
50	S_collat_transv_ant	0.003	0.079	0.035	0.034	0.955	0.081
51	S_collat_transv_post	0.062	0.059	0.035	0.035	0.208	0.223
52	S_front_inf	0.065	-0.005	0.033	0.033	0.170	0.920
53	S_front_middle	-0.018	0.001	0.036	0.034	0.788	0.986
54	S_front_sup	0.019	**0.095**	0.032	0.032	0.738	**0.017**
55	S_interm_prim-Jensen	0.012	0.029	0.036	0.036	0.864	0.619

56	S_intrapariet_and_P_trans	0.051	-0.036	0.034	0.033	0.293	0.460
57	S_oc_middle_and_Lunatus	0.000	-0.026	0.035	0.035	0.990	0.653
58	S_oc_sup_and_transversal	-0.010	-0.045	0.035	0.035	0.875	0.374
59	S_occipital_ant	0.014	0.011	0.036	0.036	0.839	0.875
60	S_oc-temp_lat	0.007	**0.108**	0.036	0.035	0.900	**0.016**
61	S_oc-temp_med_and_Lingual	0.059	0.038	0.035	0.035	0.223	0.460
62	S_orbital_lateral	-0.023	-0.061	0.036	0.035	0.716	0.220
63	S_orbital_med-olfact	-0.019	-0.018	0.036	0.036	0.786	0.786
64	S_orbital-H_Shaped	-0.004	0.024	0.029	0.029	0.920	0.618
65	S_parieto_occipital	0.033	0.028	0.035	0.034	0.550	0.618
66	S_pericallosal	-0.073	-0.053	0.031	0.031	0.069	0.223
67	S_postcentral	0.009	-0.050	0.033	0.033	0.875	0.295
68	S_precentral-inf-part	-0.029	-0.019	0.033	0.033	0.591	0.754
69	S_precentral-sup-part	0.037	0.040	0.032	0.033	0.421	0.391
70	S_suborbital	-0.023	0.009	0.034	0.035	0.699	0.875
71	S_subparietal	-0.028	0.017	0.033	0.033	0.601	0.786
72	S_temporal_inf	0.052	**0.088**	0.033	0.033	0.270	**0.039**
73	S_temporal_sup	0.037	-0.015	0.030	0.031	0.391	0.798
74	S_temporal_transverse	-0.014	-0.057	0.034	0.034	0.839	0.223

Table S3 Linear regression results for 148 cortical regions (G refers to gyrus and S refers to sulcus) via Model 2 where totally 162 regional BVs (parcellated based on Destrieux atlas) are regressed on iICAC T, controlling for age, sex, population, and total intracranial volume, after false discovery rate correction

		β_{T-std}		SE		*p*	
Index	Region	Lh	Rh	Lh	Rh	Lh	Rh
1	G_and_S_frontomargin	-0.010	-0.003	0.033	0.034	0.913	0.985
2	G_and_S_occipital_inf	0.033	-0.025	0.032	0.032	0.629	0.733
3	G_and_S_paracentral	-0.059	-0.028	0.031	0.031	0.270	0.682
4	G_and_S_subcentral	**-0.093**	-0.037	0.030	0.031	**0.037**	0.571
5	G_and_S_transv_frontopol	0.016	-0.055	0.032	0.032	0.814	0.322
6	G_and_S_cingul-Ant	0.015	0.029	0.027	0.026	0.785	0.572
7	G_and_S_cingul-Mid-Ant	0.011	0.009	0.030	0.029	0.875	0.912
8	G_and_S_cingul-Mid-Post	-0.012	0.032	0.030	0.030	0.869	0.621
9	G_cingul-Post-dorsal	-0.020	-0.025	0.031	0.031	0.761	0.732
10	G_cingul-Post-ventral	-0.027	-0.070	0.033	0.034	0.721	0.231
11	G_cuneus	0.002	0.045	0.032	0.031	0.985	0.411
12	G_front_inf-Opercular	-0.026	0.023	0.032	0.033	0.721	0.733
13	G_front_inf-Orbital	-0.030	-0.006	0.033	0.034	0.693	0.964
14	G_front_inf-Triangul	0.040	0.011	0.033	0.033	0.566	0.897
15	G_front_middle	-0.064	-0.044	0.028	0.028	0.166	0.379
16	G_front_sup	-0.003	0.023	0.027	0.027	0.979	0.709
17	G_Ins_lg_and_S_cent_ins	0.017	0.069	0.029	0.030	0.784	0.166
18	G_insular_short	-0.038	-0.007	0.029	0.030	0.521	0.952
19	G_occipital_middle	-0.023	0.007	0.032	0.033	0.733	0.955
20	G_occipital_sup	-0.002	-0.061	0.034	0.034	0.985	0.300
21	G_oc-temp_lat-fusifor	0.049	0.069	0.032	0.031	0.379	0.166
22	G_oc-temp_med-Lingual	-0.004	0.029	0.031	0.030	0.979	0.644
23	G_oc-temp_med-Parahip	0.003	-0.022	0.032	0.031	0.985	0.733
24	G_orbital	0.029	0.038	0.028	0.028	0.629	0.498
25	G_pariet_inf-Angular	-0.026	-0.042	0.032	0.031	0.721	0.496
26	G_pariet_inf-Supramar	-0.081	-0.082	0.030	0.030	0.087	0.087
27	G_parietal_sup	-0.039	-0.047	0.031	0.031	0.553	0.379
28	G_postcentral	-0.083	**-0.099**	0.030	0.030	0.087	**0.026**
29	G_precentral	**-0.099**	-0.052	0.029	0.030	**0.021**	0.322
30	G_precuneus	-0.001	-0.012	0.030	0.030	0.985	0.869
31	G_rectus	-0.025	-0.037	0.030	0.032	0.721	0.572
32	G_subcallosal	-0.042	0.026	0.031	0.030	0.481	0.693
33	G_temp_sup-G_T_transv	0.001	-0.012	0.031	0.032	0.985	0.875
34	G_temp_sup-Lateral	-0.003	-0.023	0.031	0.030	0.985	0.733
35	G_temp_sup-Plan_polar	0.056	0.005	0.029	0.028	0.270	0.960
36	G_temp_sup-Plan_tempo	0.002	0.042	0.033	0.034	0.985	0.553
37	G_temporal_inf	0.019	0.070	0.031	0.031	0.784	0.166
38	G_temporal_middle	0.021	0.054	0.029	0.030	0.733	0.300
39	Lat_Fis-ant-Horizont	0.020	-0.015	0.034	0.033	0.784	0.846
40	Lat_Fis-ant-Vertical	0.069	0.068	0.034	0.033	0.232	0.231
41	Lat_Fis-post	-0.031	0.021	0.030	0.029	0.629	0.733
42	Pole_occipital	-0.024	-0.022	0.032	0.031	0.733	0.733
43	Pole_temporal	-0.001	0.049	0.032	0.032	0.985	0.379
44	S_calcarine	0.057	0.017	0.032	0.031	0.300	0.789
45	S_central	0.001	0.030	0.031	0.032	0.985	0.671
46	S_cingul-Marginalis	-0.014	-0.003	0.032	0.032	0.848	0.985
47	S_circular_insula_ant	0.028	0.034	0.031	0.030	0.682	0.572
48	S_circular_insula_inf	0.041	**0.098**	0.026	0.027	0.379	**0.013**
49	S_circular_insula_sup	0.056	0.066	0.029	0.028	0.262	0.166
50	S_collat_transv_ant	0.054	0.076	0.034	0.033	0.358	0.166
51	S_collat_transv_post	0.023	-0.008	0.034	0.034	0.733	0.952
52	S_front_inf	0.053	0.032	0.032	0.031	0.343	0.629
53	S_front_middle	0.023	-0.040	0.034	0.033	0.733	0.559
54	S_front_sup	0.055	0.076	0.030	0.030	0.300	0.136
55	S_interm_prim-Jensen	0.039	0.057	0.034	0.034	0.572	0.343

56	S_intrapariet_and_P_trans	0.024	-0.018	0.032	0.032	0.733	0.785
57	S_oc_middle_and_Lunatus	0.002	0.009	0.033	0.033	0.985	0.940
58	S_oc_sup_and_transversal	0.023	-0.053	0.034	0.033	0.733	0.372
59	S_occipital_ant	-0.005	-0.007	0.035	0.034	0.979	0.960
60	S_oc-temp_lat	-0.005	0.028	0.035	0.034	0.979	0.721
61	S_oc-temp_med_and_Lingual	0.056	0.019	0.034	0.034	0.343	0.784
62	S_orbital_lateral	0.040	0.028	0.034	0.034	0.572	0.721
63	S_orbital_med-olfact	-0.019	-0.079	0.034	0.034	0.790	0.166
64	S_orbital-H_Shaped	0.046	0.062	0.027	0.028	0.343	0.166
65	S_parieto_occipital	**0.116**	0.053	0.033	0.033	**0.016**	0.344
66	S_pericallosal	-0.015	-0.019	0.030	0.030	0.814	0.767
67	S_postcentral	0.064	0.031	0.031	0.032	0.231	0.646
68	S_precentral-inf-part	0.019	0.011	0.032	0.031	0.784	0.894
69	S_precentral-sup-part	0.034	0.032	0.030	0.031	0.584	0.629
70	S_suborbital	0.057	0.060	0.032	0.033	0.322	0.300
71	S_subparietal	-0.001	0.015	0.032	0.032	0.985	0.814
72	S_temporal_inf	0.046	**0.115**	0.032	0.032	0.435	**0.013**
73	S_temporal_sup	0.064	0.022	0.029	0.029	0.166	0.733
74	S_temporal_transverse	0.031	-0.007	0.033	0.032	0.675	0.952

Table S4 Linear regression results for 148 cortical regions (G refers to gyrus and S refers to sulcus) via Model 3 where totally 162 regional BVs (parcellated based on Destrieux atlas) are regressed on iICAC S and T simultaneously, controlling for age, sex, population, and total intracranial volume, after false discovery rate correction

		β_{S-std}		β_{T-std}	
Index	Region	Lh	Rh	Lh	Rh
1	G_and_S_frontomargin	**-0.111**	**-0.108**	0.031	0.038
2	G_and_S_occipital_inf	-0.027	-0.008	0.043	-0.022
3	G_and_S_paracentral	-0.053	-0.060	-0.039	-0.006
4	G_and_S_subcentral	**-0.106**	**-0.119**	-0.053	0.008
5	G_and_S_transv_frontopol	**-0.143**	-0.075	0.069	-0.027
6	G_and_S_cingul-Ant	-0.012	0.002	0.020	0.028
7	G_and_S_cingul-Mid-Ant	-0.013	0.018	0.016	0.002
8	G_and_S_cingul-Mid-Post	**-0.115**	**-0.102**	0.031	0.070
9	G_cingul-Post-dorsal	**-0.094**	-0.045	0.016	-0.008
10	G_cingul-Post-ventral	-0.028	-0.016	-0.016	-0.064
11	G_cuneus	0.007	0.020	-0.001	0.038
12	G_front_inf-Opercular	-0.086	**-0.124**	0.006	0.070
13	G_front_inf-Orbital	-0.002	-0.008	-0.029	-0.003
14	G_front_inf-Triangul	-0.091	-0.074	0.074	0.039
15	G_front_middle	-0.018	-0.009	-0.057	-0.041
16	G_front_sup	-0.045	-0.012	0.013	0.028
17	G_Ins_lg_and_S_cent_ins	-0.025	-0.057	0.026	0.091
18	G_insular_short	-0.028	-0.057	-0.027	0.015
19	G_occipital_middle	-0.064	-0.055	0.001	0.027
20	G_occipital_sup	-0.088	-0.043	0.031	-0.045
21	G_oc-temp_lat-fusifor	0.021	0.042	0.041	0.053
22	G_oc-temp_med-Lingual	0.007	0.026	-0.006	0.019
23	G_oc-temp_med-Parahip	-0.013	0.037	0.007	-0.036
24	G_orbital	-0.070	-0.062	0.055	0.061
25	G_pariet_inf-Angular	-0.083	-0.068	0.005	-0.016
26	G_pariet_inf-Supramar	**-0.094**	**-0.100**	-0.046	-0.044
27	G_parietal_sup	-0.056	-0.036	-0.018	-0.033
28	G_postcentral	-0.031	-0.049	-0.071	-0.081
29	G_precentral	**-0.134**	**-0.112**	-0.049	-0.010
30	G_precuneus	-0.022	-0.004	0.007	-0.011
31	G_rectus	-0.038	**-0.093**	-0.010	-0.003
32	G_subcallosal	-0.005	-0.073	-0.040	0.054
33	G_temp_sup-G_T_transv	**-0.110**	-0.047	0.042	0.005
34	G_temp_sup-Lateral	**-0.145**	**-0.130**	0.052	0.026
35	G_temp_sup-Plan_polar	-0.018	-0.066	0.062	0.030
36	G_temp_sup-Plan_tempo	-0.076	-0.036	0.030	0.056
37	G_temporal_inf	-0.006	-0.015	0.021	0.076
38	G_temporal_middle	-0.038	-0.080	0.035	0.084
39	Lat_Fis-ant-Horizont	-0.024	-0.069	0.028	0.011
40	Lat_Fis-ant-Vertical	0.078	-0.017	0.040	0.074
41	Lat_Fis-post	**-0.099**	-0.062	0.006	0.044
42	Pole_occipital	-0.044	-0.034	-0.008	-0.009
43	Pole_temporal	**-0.104**	**-0.094**	0.038	0.084
44	S_calcarine	-0.066	0.026	0.082	0.007
45	S_central	-0.007	-0.005	0.003	0.032
46	S_cingul-Marginalis	-0.004	-0.051	-0.012	0.016
47	S_circular_insula_ant	0.067	0.038	0.003	0.020
48	S_circular_insula_inf	-0.005	-0.006	0.042	0.101
49	S_circular_insula_sup	0.035	0.054	0.042	0.046
50	S_collat_transv_ant	-0.023	0.056	0.063	0.055
51	S_collat_transv_post	0.063	0.074	0.000	-0.036
52	S_front_inf	0.051	-0.021	0.034	0.040
53	S_front_middle	-0.033	0.020	0.035	-0.047
54	S_front_sup	-0.004	0.076	0.056	0.048

55	S_interm_prim-Jensen	-0.005	0.007	0.041	0.054
56	S_intrapariet_and_P_trans	0.048	-0.034	0.006	-0.005
57	S_oc_middle_and_Lunatus	-0.001	-0.035	0.003	0.022
58	S_oc_sup_and_transversal	-0.023	-0.027	0.031	-0.043
59	S_occipital_ant	0.019	0.016	-0.012	-0.012
60	S_oc-temp_lat	0.011	**0.114**	-0.009	-0.015
61	S_oc-temp_med_and_Lingual	0.043	0.035	0.040	0.006
62	S_orbital_lateral	-0.046	-0.086	0.057	0.060
63	S_orbital_med-olfact	-0.013	0.016	-0.014	-0.085
64	S_orbital-H_Shaped	-0.027	-0.002	0.056	0.063
65	S_parieto_occipital	-0.017	0.007	0.122	0.051
66	S_pericallosal	-0.079	-0.053	0.015	0.001
67	S_postcentral	-0.020	-0.073	0.071	0.059
68	S_precentral-inf-part	-0.043	-0.028	0.035	0.021
69	S_precentral-sup-part	0.027	0.032	0.024	0.020
70	S_suborbital	-0.054	-0.018	0.077	0.067
71	S_subparietal	-0.033	0.013	0.011	0.010
72	S_temporal_inf	0.039	0.049	0.031	0.097
73	S_temporal_sup	0.013	-0.028	0.059	0.032
74	S_temporal_transverse	-0.031	-0.064	0.042	0.016

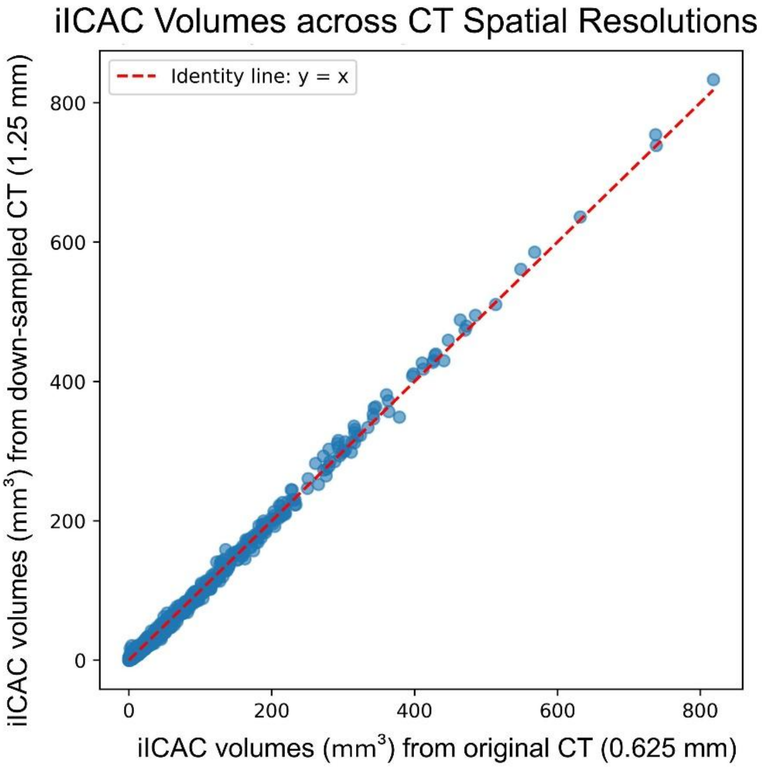

Fig. S1 Reproducibility of iICAC volumes across CT spatial resolutions. Scatter plot of iICAC volumes measured from original CT scans (0.625 mm resolution) versus down-sampled scans (1.25 mm resolution). Each point represents a participant. The red dashed line indicates the identity line (y = x)

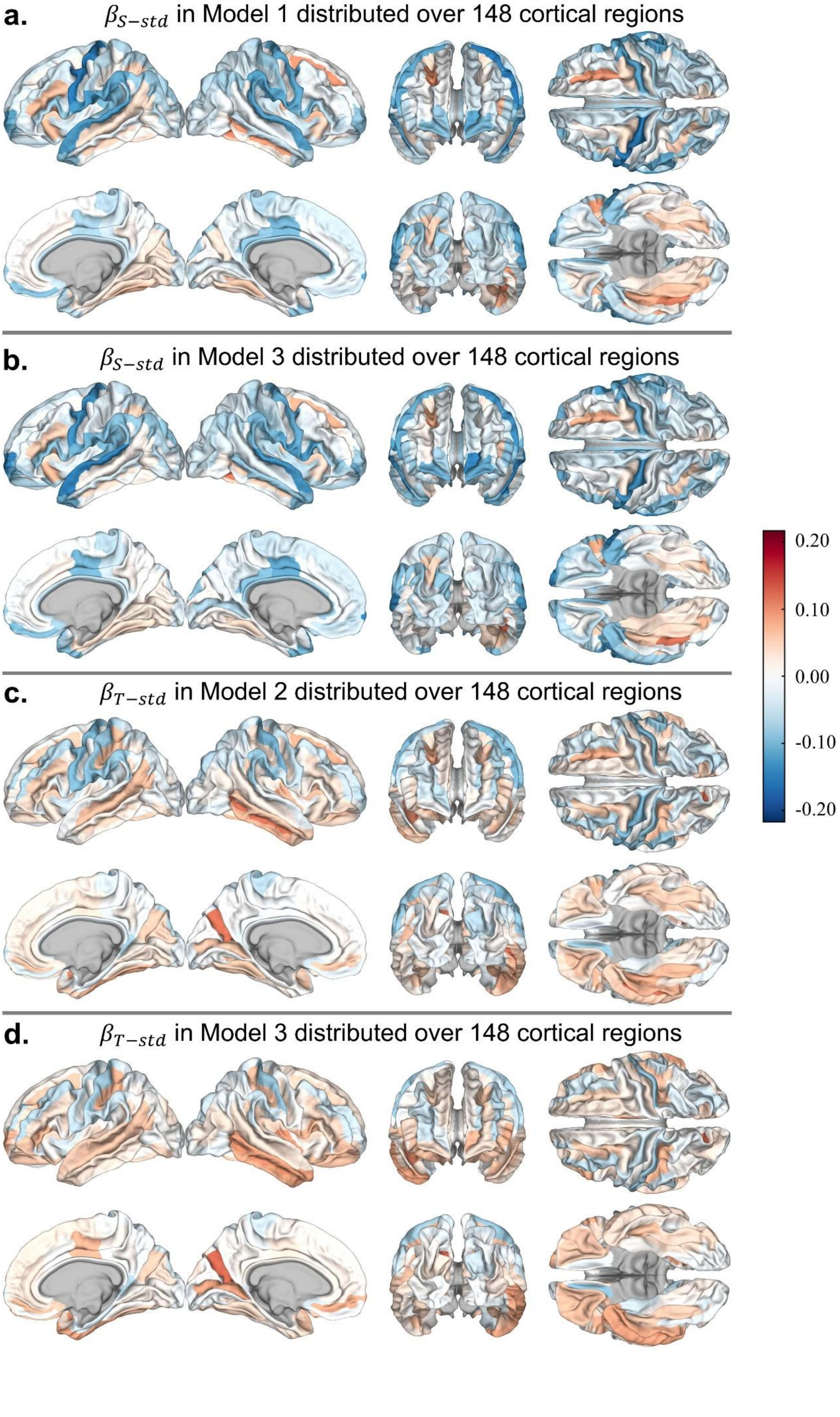
a.
β_{S-std} in Model 1 distributed over 148 cortical regions
b.
β_{S-std} in Model 3 distributed over 148 cortical regions
c.
β_{T-std} in Model 2 distributed over 148 cortical regions
d.
β_{T-std} in Model 3 distributed over 148 cortical regions
0.20
0.10
0.00
-0.10
-0.20

Fig. S2 Standardized estimates distributed over 148 cortical regions. **a** visualizes the standardized estimates of iICAC *S* (β_{S-std}s) from Model 1 where total 162 regional BVs are regressed only on iICAC *S*. **b** visualizes the standardized estimates of iICAC *S* (β_{S-std}s) from Model 3 regressed on iICAC *S* and *T* simultaneously. **c** visualizes the standardized estimates of iICAC *T* (β_{T-std}s) from Model 2 regressed only on iICAC *T*. **d** visualizes the standardized estimates of iICAC *T* (β_{T-std}s) from Model 3 regressed on iICAC *S* and *T* simultaneously. Data was analyzed by linear regression, adjusted for age, sex, population, and total intracranial volume, after false discovery rate correction

Article title:

Segmentation and morphometry of intracranial internal carotid artery calcification in relation to brain atrophy

Journal name:

Neuroradiology

Author names:

Xiao Xu, Nikhil N. Chaudhari, Phoebe Imms, Nahian F. Chowdhury, Fangyun Liu, Jorge A. Solis Galvan, Bavrina Bigjahan, Grant Schleifer, Maria Ashna, Blake Hannagan, Giuseppe Barisano, Daniel K. Cummings, Daniel Eid Rodriguez, Paul L. Hooper, Edmond Seabright, Randall C. Thompson, Benjamin C. Trumble, Michael D. Gurven, Jonathan Stieglitz, Caleb E. Finch, M. Linda Sutherland, James D. Sutherland, Helena C. Chui, Margaret Gatz, Wendy J. Mack, Hillard Kaplan, and Andrei Irimia

Corresponding author:

Andrei Irimia, Ethel Percy Andrus Center, Leonard Davis School of Gerontology, University of Southern California, Los Angeles, CA, USA

Email: irimia@usc.edu
